# Immediate skin-to-skin contact after caesarean section – A simulation study

**DOI:** 10.1016/j.eurox.2025.100392

**Published:** 2025-05-14

**Authors:** Leena Taittonen, Tanja Mäkynen, Tiina-Liisa Erkinheimo

**Affiliations:** Tampere Centre for Child, Adolescent and Maternal Health Research, Faculty of Medicine and Health Technology, Tampere University, Tampere, Finland; Department of Gynaecology, Central Hospital of Southern Ostrobothnia Hospital District, Seinäjoki, Finland

**Keywords:** Caesarean section, Descriptive study, Skin-to-skin contact

## Abstract

**Background:**

Performing a caesarean section using a drape with a transparent window between the mother and the surgical area has not been studied previously.

**Aim:**

To describe ways of passing a baby to the mother for skin-to-skin contact after caesarean section, and to analyse the time lapse between cutting the umbilical cord and placement of the baby on the mother.

**Methods:**

Simulation characters were used as study subjects. Three methods of passing a baby to the mother for skin-to-skin contact after caesarean section were assessed. In the first method, an opaque drape was placed between the mother and the surgical area, so the mother could not see the baby being born. The surgeon passed the baby to the midwife, who placed the baby on the mother for skin-to-skin contact. In the second method, the mother was able to see the baby being born through a transparent drape. The baby was otherwise handled as in the first method. In the third method, a transparent drape with a window was placed between the mother and the surgical area, so the mother could see the baby being born. The baby was passed through the window in the drape and placed on the mother for skin-to-skin contact. The time lapse between cutting the umbilical cut and skin-to-skin contact was analysed for the three methods.

**Results:**

For the first and second methods, the time between cutting the umbilical cord and skin-to-skin contact ranged from 11 to 15.5 s. For the third method, the time was 20–29 s. A minor technical difficulty was noted for the third method.

**Conclusion:**

Passing a baby to the mother through a transparent drape with a window after caesarean section is not superior in terms of time, but may improve bonding between the mother and baby.

## 1 Introduction

Gentle caesarean section has been described as a more natural mode of care for mothers and babies compared with standard caesarean section [1]. The most crucial elements of gentle caesarean section are immediate skin-to-skin contact and no separation of the mother and baby. A study in Switzerland introduced another option for gentle caesarean section, in which the mother was able to see the baby being born through use of a transparent drape separating the mother from the surgical area [2]. However, the method for passing the baby to the mother for skin-to-skin contact after cutting the umbilical cord has not been described for either method. Technically, it is possible to pass the baby to the mother through a drape with a window opening between the mother and the surgical area. This method for passing the baby to the mother may be the fastest way to achieve skin-to-skin contact between the mother and baby. Furthermore, this method may have several other positive impacts, such as improving the baby’s bacterial flora and enhancing bonding between the mother and baby. To the authors’ knowledge, no studies on this method have been undertaken to date.

The aim of this study was to analyse whether skin-to-skin contact could be achieved more quickly when a drape with a window opening was used compared with more traditional methods of passing the baby to the mother.

## 2 Methods

This study was performed in the Department of Gynaecology, Central Hospital of Southern Ostrobothnia Hospital District, Seinäjoki, Finland. No patients were involved; instead, simulation characters (mannequins) were used to assess the three methods for passing a baby to the mother for skin-to-skin contact after caesarean section. The study was performed with good ethical standards, and was approved by the medical director of the hospital. The procedures were videorecorded, and the time between cutting the umbilical cord and skin-to-skin contact was recorded. This time lapse was used as the main variable in this study.

In this study, the simulation was repeated three times for each method with the same personnel to document the three methods for passing a baby to the mother for skin-to-skin contact after caesarean section. Two different angles were used for filming. Videos from all procedures were stored, and a single synopsis video was formed from the separate recordings (Supplementary Material 1).

### 2.1 Traditional method

**Fig. 1:**
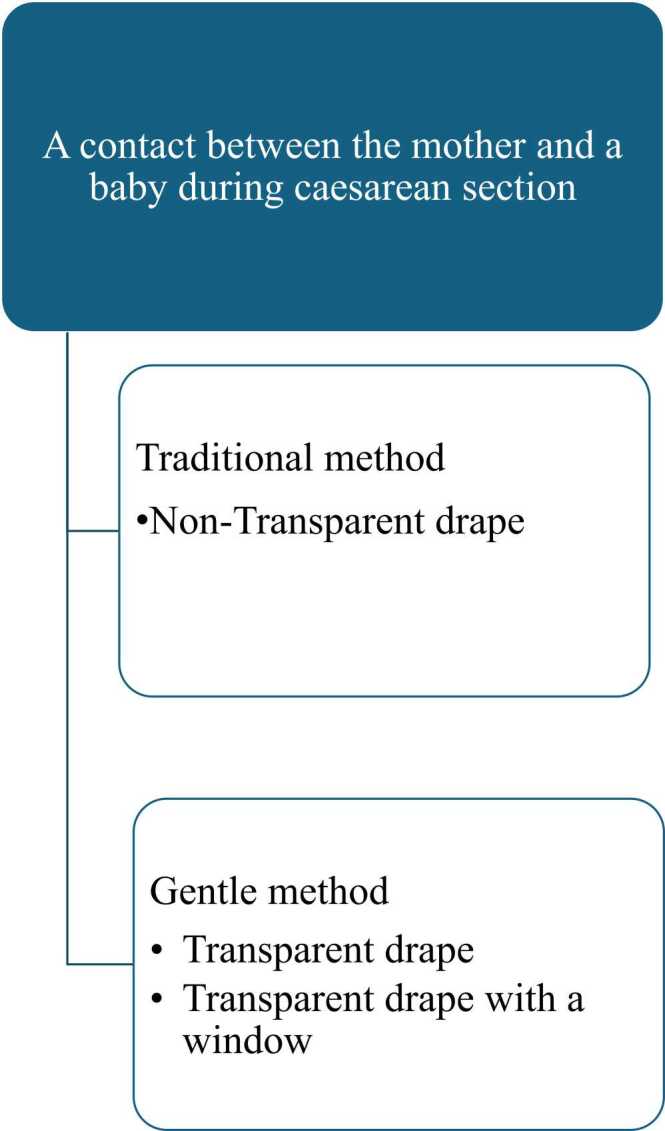
Three methods for passing a baby to the mother for skin-to-skin contact after caesarean section.

In the first method, an opaque drape was used to separate the mother from the surgical area (Fig. 1, Table 1). The drape was placed between the surgical area and mother’s upper body. The drape prevented the mother from seeing the baby being born. In this method, the baby was passed from the surgeon to the midwife, who placed the baby on the mother for skin-to-skin contact (Fig. 1, Table 1, Supplementary Material 1).

**Table 1:** Materials.

Method	Material
Traditional opaque drape	Individual c-section package, ref 97022577–10, Mölnlycke
Transparent drape	Vertical Drape Large, ref 1332–02, Evercare
Transparent drape with window	OPS Essential skin-to-skin section drape, ref MTT0523-ISK, Medline EU

### 2.2 Gentle method with a transparent drape

In the second method, a transparent plastic drape was placed between the mother and the surgical area. This enabled the mother to see the baby being born. As in the first method, the baby was passed from the surgeon to the midwife, who placed the baby on the mother for skin-to-skin contact (Fig. 1, Table 1, Supplementary Material 1).

### 2.3 Gentle method with a transparent drape with a window

In the third method, a transparent drape with a window was placed between the mother and the surgical area. The mother was able to see the baby being born. The baby was passed directly from the surgical area to the mother through the window in the drape (Fig. 1, Table 1, Supplementary Material 1).

## 3 Results

Each method for passing the baby to the mother for skin-to-skin contact is shown in the synopsis video (Supplementary Material 1). The procedure went well in all cases except one, when difficulty was experienced opening the window in the drape. This increased the time lapse between cutting the umbilical cord and placing the baby on the mother by approximately 4 s. This difficulty was not noted in the other two scenarios using the drape with a window.

For the traditional method and the transparent drape (no window) method, the time between cutting the umbilical cord and placing the baby on the mother for skin-to-skin contact was the same (11–15.5 s). For the method using a transparent drape with a window, the time between cutting the umbilical cord and placing the baby on the mother for skin-to-skin contact varied between 20 and 29 s.

## 4 Discussion

### 4.1 Main findings

To the authors’ knowledge, this is the first study to assess methods of handling a baby after caesarean section using videotaping. The three methods of passing the baby from the surgical area to the mother for skin-to-skin contact differed somewhat in terms of the time lapse between cutting the umbilical cord and skin-to-skin contact with the mother. In the first two methods, where the midwife took the baby from the surgical area to the mother, the time lapse between cutting the umbilical cord and skin-to-skin contact with the mother was less compared with the method where the baby was passed to the mother through a window in a transparent drape. The time lag for the latter method seemed to be due to placing the baby with its back towards the drape and ripping the drape window open. Difficulty was experienced when opening the drape window, but this was easily solved by the midwife forcing the window open. However, special tools may be needed to assist with opening the window. Furthermore, quality assurance of this type of drape may need improvement before widescale utilisation.

### 4.2 Strengths and limitations

A strength of this study was the repetition, in a controlled way, of passing a baby to the mother for skin-to-skin contact. The video recordings made it possible to analyse the time lapse between cutting the umbilical cord and skin-to-skin contact, and to form a synopsis video from the separate recordings.

A limitation of this study was due to the use of simulation characters instead of patients. Temperature control of the baby, which is an important prognostic variable among premature babies in particular [12], could not be evaluated using simulation characters. Furthermore, the human factors of the mother and baby could not be evaluated.

### 4.3 Interpretation

The positive experiences of gentle caesarean section reported by mothers in an earlier study [2] may have encouraged more widespread use of this method by clinicians. However, to the authors’ knowledge, no studies on the popularity of this method have been published to date. This study describes, with the help of videos, the possible methods for placement of a baby on the mother for skin-to-skin contact after caesarean section. This may improve the use of gentle caesarean section methods.

The gut microbiome is known to differ between babies born via caesarean section and babies born vaginally [3], [4]. To compensate for this, newborn babies have been swiped with a tampon with the mother’s vaginal secretions in a research setting [5]. This seeding of babies has generated discussions about its efficacy and safety [6], and the method is not currently in widespread use. Passing a baby to the mother through a window in the surgical drape, as described here, may not expose the baby to the same bacteria as vaginal delivery, but may protect the baby from hospital-originated bacteria. Theoretically, this occurs when a baby is passed directly from a sterile surgical area to their mother’s skin. In this way, the mother’s skin flora may colonise the baby’s skin.

Immediate skin-to-skin contact and early breastfeeding are two closely-linked interventions that need to take place immediately after birth, and ideally together for optimal benefit [7], [8], [9]. Immediate and prolonged skin-to-skin care facilitates breastfeeding, populates the infant’s microbiome, helps to prevent hypothermia and hypoglycaemia, stabilises respiratory function [8], and reduces mortality among low-birthweight infants [8], [9]. The first hours after birth represent a sensitive period for infants with very low birth weight, and mothers who see their premature infant within 3 h of birth are likely to establish a more secure attachment to the baby compared with those who do not see their baby within 3 h [10]. Few studies have been undertaken on the effects of bonding between mothers and babies following caesarean section [11]. In the present study, two of the methods may improve bonding compared with the traditional method, where the mother does not see the baby being born. Studies comparing these gentle caesarean methods with the traditional method in terms of short- and long-term effects have not been performed to date. In this study, although the method using a window in a transparent drape was not superior in terms of time, it may offer a way to improve maternal satisfaction and bonding between the mother and baby compared with more traditional ways of handling the baby after a caesarean section, as seen in an earlier study [2].

## 5 Conclusion and future directions

Three methods for passing a baby to the mother for skin-to-skin contact after caesarean section were analysed using videos, and a synopsis video was created.

The method using a transparent drape with a window was not superior in terms of time, but may offer other benefits for mother and child bonding. There is a need for technical reassurance regarding difficulties associated with opening the drape window before widescale utilisation.

The video showing how the baby was passed to the mother for skin-to-skin contact using simulation characters may facilitate clinical studies to investigate the opinions of mothers on the methods.

Looking at the effects of extended gentle caesarean section on bonding and breastfeeding are potential areas for further study.

## Funding

This work was supported by a grant from Vaasa Medical Association, Vaasa, Finland, and the University of Tampere Foundation sr Number of work contract 104728, Helsinki, Finland.

## Declaration of Competing Interest

We declare that the manuscript:” Immediate Skin-to-skin Contact During Caesarean Section – A Simulation Study” has not been published previously and is not under consideration for publication elsewhere. The article’s publication is approved by all authors. If accepted the article will not be published elsewhere in the same form, in English or in any other language, including electronically, without the written consent of the copyright-holder.

## Appendix A Supplementary material

**Supplementary Material 1**. **Synopsis video of the three methods for passing a baby to the mother for skin-to-skin contact after caesarean section**
